# Interaction sites of DivIVA and RodA from *Corynebacterium glutamicum*

**DOI:** 10.3389/fmicb.2014.00738

**Published:** 2015-01-07

**Authors:** Boris Sieger, Marc Bramkamp

**Affiliations:** Biocenter – Ludwig-Maximilians-University MunichMunich, Germany

**Keywords:** DivIVA, RodA, *Corynebacterium glutamicum*, protein–protein interactions, polar cell growth, FRET

## Abstract

Elongation growth in actinobacteria is localized at the cell poles. This is in contrast to many classical model organisms where insertion of new cell wall material is localized around the lateral site. We previously described a role of RodA from *Corynebacterium glutamicum* in apical cell growth and morphogenesis. Deletion of *rodA* had drastic effects on morphology and growth, likely a result from misregulation of penicillin-binding proteins and cell wall precursor delivery. We identified the interaction of RodA with the polar scaffold protein DivIVA, thus explaining subcellular localization of RodA to the cell poles. In this study, we describe this interaction in detail and map the interaction sites of DivIVA and RodA. A single amino acid residue in the N-terminal domain of DivIVA was found to be crucial for the interaction with RodA. The interaction site of RodA was mapped to its cytoplasmic, C-terminal domain, in a region encompassing the last 10 amino acids (AAs). Deletion of these 10 AAs significantly decreased the interaction efficiency with DivIVA. Our results corroborate the interaction of DivIVA and RodA, underscoring the important role of DivIVA as a spatial organizer of the elongation machinery in *Corynebacterineae*.

## INTRODUCTION

*Corynebacterium glutamicum* is a fast growing, facultative anaerobic, Gram positive *Actinobacterium* with high industrial importance in the production of amino acids (AAs; Ikeda and Nakagawa, 2003). For this purpose, fast growth rates and high cell densities are two major properties to ensure efficient production rates. Furthermore, *C. glutamicum* gained medical interest due to its mycobacteria-like cell wall and its phylogenetic relation to notorious pathogens such as *C. diphtheriae*, *Mycobacterium tuberculosis,* and *M. leprae* (Barh et al., 2011; Cayabyab et al., 2012). Members of the genus *Corynebacterium* are abundant species on the human skin and airways microbiome (Cowling and Hall, 1993; Zeeuwen et al., 2013). *C. glutamicum* and all other actinobacteria grow apically by insertion of new cell wall material at the cell poles (Locci and Schaal, 1980; Brown et al., 2011; Donovan and Bramkamp, 2014). Spatial localization of the cell wall machinery is governed by a coiled-coil protein, DivIVA (Letek et al., 2008; Sieger et al., 2013). This is in remarkable contrast to rod-shaped bacteria from other phyla such as firmicutes or proteobacteria. In these organisms a MreBCD-based cell wall synthetic machinery is acting along the lateral sites of the cell (Jones et al., 2001; Kruse et al., 2005). The processive enzymes, e.g. penicillin-binding proteins are, however, ubiquitous (Popham and Young, 2003). Furthermore, membrane integral proteins of the SEDS family are found in every cell wall synthetic cluster (Henriques et al., 1998; Pastoret et al., 2004). It is believed that an FtsW homolog is associated with septal cell wall synthesis, while RodA homologs are part of the elongation machinery (Pastoret et al., 2004; Real et al., 2008; Sieger et al., 2013). FtsW and RodA are associated with flipping the cell wall precursor lipid II (Ikeda and Nakagawa, 2003; Noirclerc-Savoye et al., 2003; Mohammadi et al., 2011). A new candidate for lipid II flipping, MurJ, has recently been described (Sham et al., 2014). Maybe, both enzymes may confer translocation of lipid II. However, based on earlier studies with *rodA* deletion mutants in *C. glutamicum*, it became evident that lack of RodA may also influence activity of the cognitive penicillin-binding proteins (Sieger et al., 2013).

**FIGURE 1:**
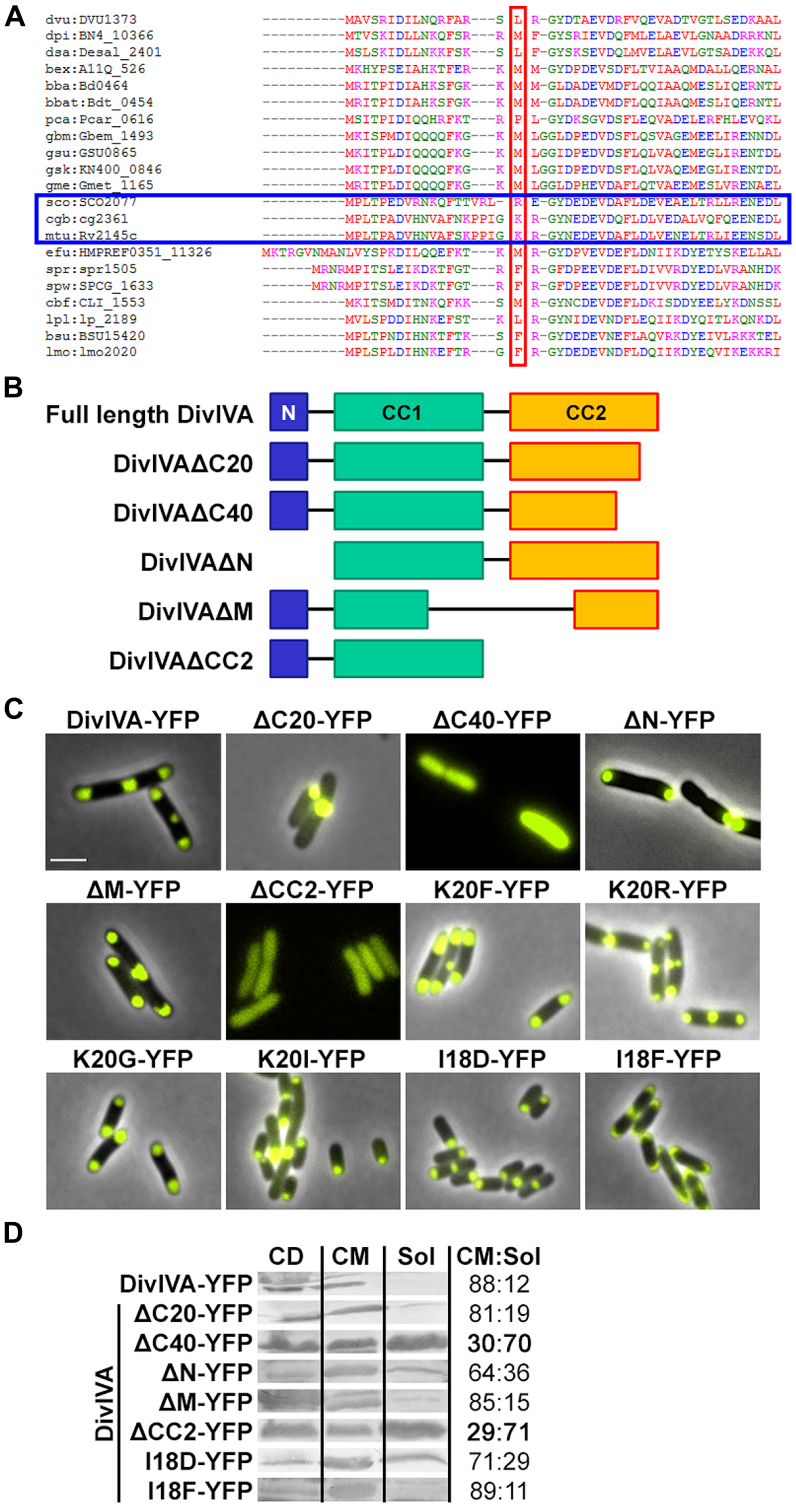
**(A)** Sequence alignment of N-terminal domains of several DivIVA proteins. Marked in red are AA residues that correspond to the F17 residue located at the tip of intertwined loops, according to the *Bacillus subtilis* DivIVA crystal structure. Polar growing actinobacteria such as *Streptomyces*, *Corynebacterium,* and *Mycobacteria* have a positively charged residue at this position (marked with a blue box). In the case of *C. glutamicum*, the corresponding AA is the lysine K20. Dvu: *Desulfovibrio vulgaris*, Dpi: *Desulfovibrio piezophilus*, Dsa: *Desulfovibrio salexigens*, Bex: *Bdellovibrio exovorus*, Bba: *Bdellovibrio bacteriovorus*, Bbat: *Bdellovibrio bacteriovorus* Tiberius, Pca: *Pelobacter carbinolicus*, Gbm: *Geobacter bemidjiensis*, Gsu: *G. sulfurreducens* PCA, Gsk: *G. sulfurreducens* KN400, Gme: *G. metallireducens*, Sco: *Streptomyces coelicolor*, Cgb: *Corynebacterium glutamicum*, Mtu: *Mycobacterium tuberculosis*, Efu: *Enterococcus faecium* DO, Spr: *Streptococcus pneumoniae* R6, Spw: *S. pneumoniae* CGSP14, Cbf: *Clostridium botulinum* F, Lpl: *Lactobacillus plantarum* WCFS1, Bsu: *B. subtilis*, Lmo: *Listeria monocytogenes*. Sequences were obtained from KEGG database. **(B)** Topology models of full length DivIVA and several truncation mutants that were generated and used in this study. DivIVAΔC20 and DivIVAΔC40 lack 20 or 40 AAs of their C-terminal ends. DivIVAΔN lacks the N-terminal domain, DivIVAΔM lacks a middle part of 154 AAs (144-298) and DivIVACC2 lacks the second coiled coil domain. **(C)** Fluorescence microscopy images of DivIVA variants that were heterologously expressed in *Escherichia coli*. Full length DivIVA-YFP localizes to both cell poles and the septum. DivIVAΔC20-YFP lost its proper localization character and forms huge, presumably non-functional, aggregates at mostly one cell pole. DivIVAΔC40-YFP as well as DivIVACC2-YFP localize in the cytoplasm, likely due to misfolding or lack of oligomerization. DivIVAΔN-YFP localizes to the poles; however, not always to both cell poles. DivIVAΔM-YFP localizes similar to wild type DivIVA. The DivIVA point mutants K20F and K20R localize similar compared to wild type protein. Mutants K20G-YFP and K20I-YFP showed polar localization, partly to one pole, comparable to DivIVAΔN-YFP. Mutant I18D-YFP showed no alterations in localization and membrane binding, whereas mutant I18F-YFP gives rise to increased membrane association. I18 is likely involved in membrane binding due to its hydrophobic character. **(D)** Ratios of non-aggregated, membrane attached vs. soluble DivIVA mutants. Full length DivIVA-YFP is 88% membrane attached and similar values were obtained from DivIVAΔC20-YFP and DivIVAΔM-YFP. DivIVAΔN-YFP is 64% membrane attached, implicating defects in membrane attachment that were drastically reduced for DivIVAΔC40-YFP and DivIVACC2-YFP to 30% and 29%, respectively. Mutant I18D-YFP had a minor decrease in membrane binding (71%) whereas I18F-YFP had a similar membrane affinity compared to WT DivIVA-YFP (89%). CD, cell debris fraction; CM, cell membrane fraction; Sol, soluble fraction. All numbers are mean values of at least three independent experiments.

In a previous study we identified RodA as being essential for growth and determination of cell shape (Sieger et al., 2013). DivIVA is frequently present in Gram positive species and generally composed of a highly conserved N-terminal domain, followed by two coiled-coil domains (Letek et al., 2009; **Figures 1A,B**). The N-terminal domain is involved in membrane attachment via exposed phenylalanine residues, positioned at the tip of intertwined loops as revealed by the crystal structure of the *Bacillus subtilis* DivIVA (Oliva et al., 2010). The coiled-coil domains are required for oligomerization and scaffold formation (Stahlberg et al., 2004; Lenarcic et al., 2009). DivIVA proteins lack an enzymatic function and polymerize into large oligomers in a nucleotide independent fashion (Muchova et al., 2002).

In the past decade several interaction partners of DivIVA have been identified. *B. subtilis* DivIVA anchors the Min system via MinJ to the cell poles, thus contributing to division site selection (Bramkamp et al., 2008; Patrick and Kearns, 2008). In addition, DivIVA interacts with RacA to attach the DNA to the prespore pole during *B. subtilis* sporulation (Ben-Yehuda et al., 2003; Wu and Errington, 2003). Further interaction partners are the division inhibitor Maf in competent *B. subtilis* cells (Briley et al., 2011), the transcriptional regulator ComN during promotion of natural competence (dos Santos et al., 2012) and SpoIIE for asymmetric division during sporulation (Eswaramoorthy et al., 2014). Only recently, it was demonstrated how MinJ and RacA of *B. subtilis* bind to separate domains of DivIVA (van Baarle et al., 2013). RacA interacts with the 11 C-terminal AAs of DivIVA, whereas MinJ binds to the N-terminal lipid binding domain. Although many more interaction partners of DivIVA have been identified in several organisms, as reviewed by (Lin and Thanbichler, 2013; Laloux and Jacobs-Wagner, 2014), little is known about their interaction sites involved in protein–protein interaction.

Here we describe the identification of the RodA–DivIVA interaction sites, thereby corroborating earlier results that suggested an interaction of *C. glutamicum* DivIVA and RodA (Sieger et al., 2013). Mutational analysis and subsequent interaction studies using a Förster-Resonance Energy Transfer (FRET)-based assay reveals that DivIVA interacts with the C-terminal domain of RodA. The N-terminal domain, which is supposed to play a role in membrane association, is crucial for the interaction with RodA. The molecular mechanism seems to include electrostatic interactions, since a positive charge in DivIVA is essential for full protein–protein interaction.

## EXPERIMENTAL PROCEDURES

### GENERAL CLONING TECHNIQUES

General cloning was performed as described before (Sieger et al., 2013). Oligonucleotides were obtained from Sigma Aldrich and are listed in Table S1. Plasmids generated in this study are listed in **Table 1**. *C. glutamicum* strains are listed in **Table 2**. *Escherichia coli* strains that were transformed with the plasmids from **Table 1** for cloning and protein expression are not listed separately. Point mutants of DivIVA were generated via overlapping PCR using oligonucleotides carrying the desired point mutation. *E. coli* was grown in Luria Broth supplemented with 100 mg ml^-1^ carbenicillin (pETDuet-1) or 50 mg ml^-1^ kanamycin (pEKEX2). *C. glutamicum* was grown in Brain Heart Infusion (BHI; Oxoid) supplemented with 25 mg ml^-1^ kanamycin (pEKEX2). Protein expression was induced with 0.1 mM IPTG.

**Table 1:** Plasmids.

No	Name	Description	Reference
**pETDuet-1**
EX001	pETDuet-1	*bla, PT7lacI-, PT7lacI-*	Novagen
BS001	Duet CFP	*bla, PT7lacI-eCFP, PT7lacI-*	Sieger et al. (2013)
BS002	Duet YFP	*bla, PT7lacI-, PT7lacI-eYFP*	Sieger et al. (2013)
BS003	Duet CFP YFP	*bla, PT7lacI-eCFP, PT7lacI-eYFP*	Sieger et al. (2013)
BS004	Duet DivIVA-YFP	*bla, PT7lacI-, PT7lacI-divIVA-eYFP*	Sieger et al. (2013)
BS005	Duet RodA-CFP	*bla, PT7lacI-rodA-eCFP, PT7lacI-*	Sieger et al. (2013)
BS006	Duet FtsW-CFP	*bla, PT7lacI-ftsW-eCFP, PT7lacI-*	Sieger et al. (2013)
BS007	Duet RodA-CFP DivIVA-YFP	*bla, PT7lacI-rodA-eCFP, PT7lacI-divIVA-eYFP*	Sieger et al. (2013)
BS008	Duet FtsW-CFP DivIVA-YFP	*bla, PT7lacI-ftsW-eCFP, PT7lacI-divIVA-eYFP*	Sieger et al. (2013)
BS025	Duet RodAΔC10-CFP	*bla, PT7lacI-rodAΔC10-eCFP PT7lacI-*	This study
BS026	Duet RodAΔC80-CFP	*bla, PT7lacI-rodAΔC80-eCFP, PT7lacI-*	This study
BS027	Duet RodA1/2-CFP	*bla, PT7lacI-rodA1/2-eCFP, PT7lacI-*	This study
BS028	Duet RodA2/2-CFP	*bla, PT7lacI-rodA2/2-eCFP, PT7lacI-*	This study
BS029	Duet RodAΔC10-CFP DivIVA-YFP	*bla, PT7lacI-rodA ΔC10-eCFP, PT7lacI-divIVA-eYFP*	This study
BS030	Duet RodAΔC80-CFP DivIVA-YFP	*bla, PT7lacI-rodA ΔC80-eCFP, PT7lacI-divIVA-eYFP*	This study
BS031	Duet RodA1/2-CFP DivIVA-YFP	*bla, PT7lacI-rodA1/2-eCFP, PT7lacI-divIVA-eYFP*	This study
BS032	Duet RodA2/2-CFP DivIVA-YFP	*bla, PT7lacI-rodA2/2-eCFP, PT7lacI-divIVA-eYFP*	This study
BS033	Duet DivIVAΔC20-YFP	*bla, PT7lacI-, PT7lacI-divIVAΔC20-eYFP*	This study
BS034	Duet DivIVAΔC40-YFP	*bla, PT7lacI-, PT7lacI-divIVAΔC40-eYFP*	This study
BS035	Duet DivIVAΔM-YFP	*bla, PT7lacI-, PT7lacI-divIVAΔM-eYFP*	This study
BS036	Duet DivIVAΔN-YFP	*bla, PT7lacI-, PT7lacI-divIVAΔN-eYFP*	This study
BS037	Duet DivIVAΔCC2-YFP	*bla, PT7lacI-, PT7lacI-divIVAΔCC2-eYFP*	This study
BS038	Duet RodA-CFP DivIVAΔC20-YFP	*bla, PT7lacI-rodA-eCFP, PT7lacI-divIVAΔC20-eYFP*	This study
BS039	Duet RodA-CFP DivIVAΔC40-YFP	*bla, PT7lacI-rodA-eCFP, PT7lacI-divIVAΔC40-eYFP*	This study
BS040	Duet RodA-CFP DivIVAΔM-YFP	*bla, PT7lacI-rodA-eCFP, PT7lacI-divIVAΔM-eYFP*	This study
BS041	Duet RodA-CFP DivIVAΔN-YFP	*bla, PT7lacI-rodA-eCFP, PT7lacI-divIVAΔN-eYFP*	This study
BS042	Duet RodA-CFP DivIVAΔCC2-YFP	*bla, PT7lacI-rodA-eCFP, PT7lacI-divIVAΔCC2-eYFP*	This study
BS043	Duet RodAE438G-CFP	*bla, PT7lacI-rodAE438G-eCFP, PT7lacI-*	This study
BS044	Duet RodAE438G-CFP DivIVA-YFP	*bla, PT7lacI-rodAE438G-eCFP, PT7lacI-divIVA-eYFP*	This study
BS045	Duet RodAK434G-CFP	*bla, PT7lacI-rodAK434G-eCFP, PT7lacI-*	This study
BS046	Duet RodAK434G-CFP, DivIVA-YFP	*bla, PT7lacI- rodAK434G-eCFP, PT7lacI-divIVA-eYFP*	This study
BS047	Duet RodAQ435G-CFP	*bla, PT7lacI-rodAQ435G-eCFP, PT7lacI-*	This study
BS048	Duet RodAQ435G-CFP, DivIVA-YFP	*bla, PT7lacI-rodAQ435G-eCFP, PT7lacI- divIVA-eYFP*	This study
BS049	Duet RodAS433G-S437G-CFP	*bla, PT7lacI-rodAS433G-S437G-eCFP, PT7lacI-*	This study
BS050	Duet RodAS433G-S437G-CFP, DivIVA-YFP	*bla, PT7lacI-rodAS433G S437G-eCFP, PT7lacI-divIVA-eYFP*	This study
BS051	Duet RodAmutC10-CFP	*bla, PT7lacI-mutC10-eCFP, PT7lacI-*	This study
BS052	Duet RodAmutC10-CFP, DivIVA-YFP	*bla, PT7lacI- mutC10-eCFP, PT7lacI-divIVA-eYFP*	This study
BS053	Duet DivIVAK20F-YFP	*bla, PT7lacI-, PT7lacI-divIVAK20F-eYFP*	This study
BS054	Duet RodA-CFP, DivIVAK20F-YFP	*bla, PT7lacI-rodA-eCFP, PT7lacI-divIVAK20F-eYFP*	This study
BS055	Duet DivIVAK20R-YFP	*bla, PT7lacI-, PT7lacI-divIVAK20R-eYFP*	This study
BS056	Duet RodA-CFP, DivIVAK20R-YFP	*bla, PT7lacI-rodA-eCFP, PT7lacI-divIVAK20R-eYFP*	This study
BS057	Duet DivIVAK20G-YFP	*bla, PT7lacI-, PT7lacI-divIVAK20G-eYFP*	This study
BS058	Duet RodA-CFP, DivIVAK20G-YFP	*bla, PT7lacI-rodA-eCFP, PT7lacI-divIVAK20G-eYFP*	This study
BS059	Duet DivIVAK20I-YFP	*bla, PT7lacI-, PT7lacI-divIVAK20I-eYFP*	This study
BS060	Duet RodA-CFP, DivIVAK20I-YFP	*bla, PT7lacI-rodA-eCFP, PT7lacI-divIVAK20I-eYFP*	This study
BS061	Duet DivIVAI18D-YFP	*bla, PT7lacI-, PT7lacI-divIVAI18D-eYFP*	This study
BS062	Duet RodA-CFP, DivIVAI18D-YFP	*bla, PT7lacI-rodA-eCFP, PT7lacI-divIVAI18D-eYFP*	This study
BS063	Duet DivIVAI18F-YFP	*bla, PT7lacI-, PT7lacI-divIVAI18F-eYFP*	This study
BS064	Duet RodA-CFP, DivIVAI18F-YFP	*bla, PT7lacI-rodA-eCFP, PT7lacI-divIVAI18F-eYFP*	This study
	**pEKEX2**
EX010	pEXEK2	*Escherichia coli* – *C. glutamicum* shuttle vector, Kan^R^, P*_tac_ lacI*^q^, pBL1 *oriV_C_*_._*_g_*_._, pUC18 *oriV_E_*_._*_c_*_._,	Eikmanns et al. (1991)
BS018	pEX2 RodA-GFP	Kan^R^, P*_tac_ lacI*^q^, pBL1 *oriV_C_*_._*_g_*_._, pUC18 *oriV_E_*_._*_c_*_._, *rodA-gfp*	Sieger et al. (2013)
BS053	pEX2 RodA1/2-GFP	Kan^R^, P*_tac_ lacI*^q^, pBL1 *oriV_C_*_._*_g_*_._, pUC18 *oriV_E_*_._*_c_*_._, *rodA1/2-gfp*	This study
BS054	pEX2 RodA2/2-GFP	Kan^R^, P*_tac_ lacI*^q^, pBL1 *oriV_C_*_._*_g_*_._, pUC18 *oriV_E_*_._*_c_*_._, *rodA2/2-gfp*	This study
BS055	pEX2 RodAΔC10-GFP	Kan^R^, P*_tac_ lacI*^q^, pBL1 *oriV_C_*_._*_g_*_._, pUC18 *oriV_E_*_._*_c_*_._, *rodAΔC10-gfp*	This study
BS056	pEX2 RodAΔC80-GFP	Kan^R^, P*_tac_ lacI*^q^, pBL1 *oriV_C_*_._*_g_*_._, pUC18 *oriV_E_*_._*_c_*_._, *rodAΔC80-gfp*	This study

**Table 2:** Strains.

Number	Genotype/Description	Reference
***C.glutamicum***
WT	ATCC 13032	Laboratory stock
Res 167	restriction deficient *C. glutamicum* mutant, otherwise considered WT	Tauch et al. (2002)
BSC001	WT, Δ*rodA*	Sieger et al. (2013)
BSC002	WT, DivIVA-mCherry, Δ*rodA*	Sieger et al. (2013)
BSC014	WT, Δ*rodA*, DivIVA-mCherry, carrying plasmid BS018	This study
BSC015	WT, Δ*rodA*, DivIVA-mCherry, carrying plasmid BS055	This study
BSC016	WT, Δ*rodA*, DivIVA-mCherry, carrying plasmid BS056	This study
BSC017	WT, Δ*rodA*, DivIVA-mCherry, carrying plasmid BS053	This study
BSC018	WT, Δ*rodA*, DivIVA-mCherry, carrying plasmid BS054	This study
***E.coli***
DH5α	F^-^ φ80*lac*Z.M15(*lac*ZYA-*arg*F)U169 *rec*A1 *end*A1 *hsd*R17(r_k_-, m_k_+) *pho*A *sup*E44 *thi*-1 *gyr*A96 *rel*A1 λ^-^	Invitrogen
BL21 (DE3)	F^-^ *ompT gal dcm lon hsdS_B_*(r*_B_*^-^ m*_B_*^-^) *λ*(DE3 [*lacI lacUV5*-T7gene1 *ind1 sam7 nin5*])	Invitrogen

### MICROSCOPY

Microscopy was performed on a Zeiss Axio Observer Z1 microscope equipped with a Hamamatsu OrcaR^2^ camera. A Plan-Apochromat 100x/1.4 Oil Ph3 objective (Zeiss) was used. YFP fluorescence was visualized with filter set 46 HE YFP shift free and CFP fluorescence with filter set 47 HE CFP shift free (Zeiss). Images were acquired with Zen software (Zeiss) or AxioVision 4.6 (Zeiss) and processed with Adobe Photoshop.

### FRET

Quantitative FRET values (R_CY_) were calculated as ratios from emission maxima of eCFP (480 nm) and eYFP (525 nm). FRET measurements were performed in late exponential growing *E. coli* cells after one washing step with 0.9% NaCl. 150 μl of cell suspension were loaded into a 96 well microtiter plate and subsequently measured in a Tecan Infinite M200 Pro plate reader. The excitation wavelength was 435 nm; emission was monitored in a range from 466 to 610 nm in 3 nm increments.

### CELL FRACTIONATION

Analysis of protein localization was performed by cell fragmentation and subsequent centrifugation. Cells were lysed in a FastPrep homogenizer (MP) in five rounds at 5 ms^-1^. Cell debris and aggregated proteins were removed by centrifugation at 14000 ×*g* for 20 min and cell membranes were harvested at 90000 ×*g* for 30 min. Together with the supernatant, samples were run on SDS PAGE and analyzed by immune-blotting. DivIVA-YFP mutants were blotted with an α-GFP antibody and visualized using an α-rabbit-alkaline phosphatase secondary antibody.

## RESULTS

### TRUNCATION MUTANTS OF DivIVA REVEAL DISTINCT DOMAIN FUNCTIONS

Several truncation mutants of DivIVA were heterologously expressed to analyze importance of the individual domains for protein localization and protein–protein interactions. *C. glutamicum* DivIVA is composed of a short N-terminal domain (N) and two coiled-coil domains (CC1 and CC2; **Figure 1B**). The topology and subcellular localization of the mutants are shown in **Figure 1C**. It turned out that CC2 is responsible for proper folding or assembly of DivIVA oligomers, as deletion of 20 AAs (ΔC20) resulted in aggregation of a likely non-functional protein and deletion of 40 AAs (ΔC40) resulted in cytoplasmic appearance. Since CC2 has been reported to play a role in oligomerization in the *B. subtilis* DivIVA (Oliva et al., 2010), this would explain how DivIVA monomers lose their ability to localize to the cell poles. Deletion of the N-terminal domain had only a minor effect, where some cell poles were free of protein, likely due to reduced membrane attachment (**Figure 1D**). 64% of the DivIVA variant ΔN was still membrane associated in contrast to 88% of the wild type protein. Deletion of a middle part of DivIVA (ΔM, AA 144-298) had no effect on DivIVA localization and deletion of CC2 showed the same localization defect as ΔC40. Cell lysate fractionation confirmed the observation that DivIVA-ΔC40 and DivIVA-ΔCC2 fail to localize to the membrane. In these two mutants only 30% (ΔC40)/29% (ΔCC2) of non-aggregated protein remains membrane attached. Full length DivIVA-YFP, ΔC20, and ΔM are 88%/81%/85%, membrane associated (**Figure 1D**).

Data derived from the crystal structure of the *B. subtilis* DivIVA suggested a role for phenylalanine (F17, *B. subtilis* numbering) in membrane-binding. This residue is not conserved in most other species (**Figure 1A**). Members of the class *Actinobacteria* possess a positively charged residue (arginine, lysine) at the corresponding position (K20 in *C. glutamicum* and *M. tuberculosis*). Sequence alignments between various DivIVA sequences reveal that the actinobacterial DivIVA homologs contain sequence insertions compared to other DivIVA sequences and it may be that other hydrophobic AAs such as isoleucine at position 18 (*C. glutamicum* numbering) might fulfill a function analogous to the F17 from *B. subtilis* (**Figure 1A**). To approach this idea, I18 was mutated to an aspartate and a phenylalanine and both mutants were tested for membrane attachment. It turned out that I18D revealed a slight decrease in membrane binding (71%), whereas I18F had a similar membrane affinity compared to WT (89%; **Figure 1D**). Finally, we mutated the lysine residue situated at position 20 in *C. glutamicum* to analyze influence of localization and DivIVA–RodA interaction. The DivIVA variants K20F and K20R localized properly at the poles and septa (**Figure 1C**), suggesting that K20 is not essential for membrane binding.

### PROTEIN–PROTEIN INTERACTIONS MEASURED BY FÖRSTER-RESONANCE ENERGY TRANSFER (FRET)

We established a FRET assay which allows visualization of protein–protein interaction in cell cultures after heterologous expression in *E. coli*. The proteins of interest are fused to CFP or YFP, respectively and expressed from plasmid pET-Duet1 (Novagen), which harbors two multiple cloning sites with two individual T7 promoters. **Table 3** shows ratios of emission maxima measured for several strains. Ratios were calculated by dividing emission maxima for CFP (525 nm) and YFP (480 nm), respectively; thereby giving a R_CY_ value that allows to judge about putative protein–protein interactions. We grouped the ratios into steps as indicated in **Table 3**, which illustrate the interaction situation of several proteins analyzed *in vivo*. R_CY_ values below 0.9 reflect a high CFP emission and low YFP emission, as obtained from strains expressing CFP or CFP-tagged proteins alone. A strain expressing soluble CFP and YFP also falls in this R_CY_ range (0.64) and demonstrates the reliability of the assay (Figure S1). Both fluorophores are evenly distributed in the cytosol; however, a FRET signal is not generated even under conditions of extreme overexpression. Values between 0.9 and 1.1 occur when CFP and YFP fluorescent fusions are present in the cell, where some of the fluorescence energy can be transferred from donor to acceptor upon random approximation of the fluorophores. This approximation can be due to topological circumstances, as it is the case for DivIVA and FtsW, a divisome specific RodA homolog. Similar ratios were obtained from other non-interacting examples such as DivIVA with BetP (a betaine carrier) or several RodA and DivIVA mutants (**Table 3**). In addition, a FRET-based ATP-sensor protein expressed from plasmid pRSETB AT1.03 (Imamura et al., 2009) served as a positive control. The ATP-sensor gave a strong FRET signal under physiological ATP levels (*R*_CY_ = 1.25). However, when the pRSETB AT1.03 cells were treated with CCCP to depolarize the membrane and thus reduce the ATP level in the cell, the R_CY_ values are 0.98 and are indicative for a loss of FRET. As another control we tested a previously described interaction partner of DivIVA, wild type ParB, and a non-interacting point mutant, ParBR21A. (Donovan et al., 2012). R_CY_ values were 1.16 for DivIVA–ParB interaction and 0.95 for DivIVA and ParBR21A (Figure S2; **Table 3**). Finally, when only YFP or YFP-tagged proteins were expressed, the emission spectra lack a CFP signal resulting in high R_CY_ values (>1.3).

**Table 3:** Classification and apportioning of FRET ratios into subgroups representing the interaction situation of the fusion proteins.

CFP/YFP ratio	Meaning	Examples			Reference
		Donor	Acceptor	Ratio	
<0.9	CFP fluorescence; no FRET	CFP	–	0.55	This study
		ParB-CFP	–	0.57	This study
		ParBR21A-CFP	–	0.57	This study
		RodA-CFP	–	0.60	Sieger et al. (2013)
		FtsW-CFP	–	0.62	Sieger et al. (2013)
		CFP	YFP	0.64	This study
		RodAmutC10-CFP	–	0.67	This study
		BetP-CFP	–	0.68	Sieger et al. (2013)
		RodAS433GS437G-CFP	–	0.68	This study
		RodAΔC10-CFP	–	0.73	This study
		RodAK434G-CFP	–	0.75	This study
		RodAQ435G-CFP	–	0.89	This study
0.9–1.1	Approximation of fluorophores; no enrichment or interaction	RodA-CFP	DivIVAΔC20-YFP	0.92	This study
		RodA-CFP	DivIVAK20F-YFP	0.93	This study
		ParBR21A-CFP	DivIVA-YFP	0.95	This study
		RodA-CFP	DivIVAK20G-YFP	0.96	This study
		RodA-CFP	DivIVAK20I-YFP	0.97	This study
		RodA-CFP	DivIVAΔN-YFP	0.97	This study
		pRSETB AT1.03	+CCCP (1 μg/ml)	0.98	Sieger et al. (2013)
		BetP-CFP	DivIVA-YFP	0.99	Sieger et al. (2013)
		RodAΔC80-CFP	DivIVA-YFP	0.99	This study
		RodAΔC10-CFP	DivIVA-YFP	1.03	This study
		RodAK434G-CFP	DivIVA-YFP	1.04	This study
		RodAQ435G-CFP	DivIVA-YFP	1.04	This study
		RodAS433GS437G-CFP	DivIVA-YFP	1.06	This study
		FtsW-CFP	DivIVA-YFP	1.09	Sieger et al. (2013)
1.1–1.3	Enrichment/interaction	RodAE438G-CFP	DivIVA-YFP	1.10	This study
		RodA-CFP	DivIVAK20R-YFP	1.11	This study
		RodA-CFP	DivIVAI18D-YFP	1.14	This study
		RodA-CFP	DivIVA-YFP	1.15	Sieger et al. (2013)
		ParB-CFP	DivIVA-YFP	1.16	This study
		RodAmutC10-CFP	DivIVA-YFP	1.17	This study
		RodA-CFP	DivIVAΔM-YFP	1.19	This study
		pRSETB AT1.03	- CCCP	1.25	Sieger et al. (2013)
		RodA-CFP	DivIVAI18F-YFP	1.27	This study
>1.3	YFP fluorescence; no FRET	–	YFP	2.55	This study
		–	DivIVA-YFP	4.43	This study
		–	DivIVAΔN-YFP	4.52	This study

### A POSITIVELY CHARGED AMINO ACID IN THE N-TERMINUS OF DivIVA IS CRUCIAL FOR INTERACTION WITH RodA

**FIGURE 2:**
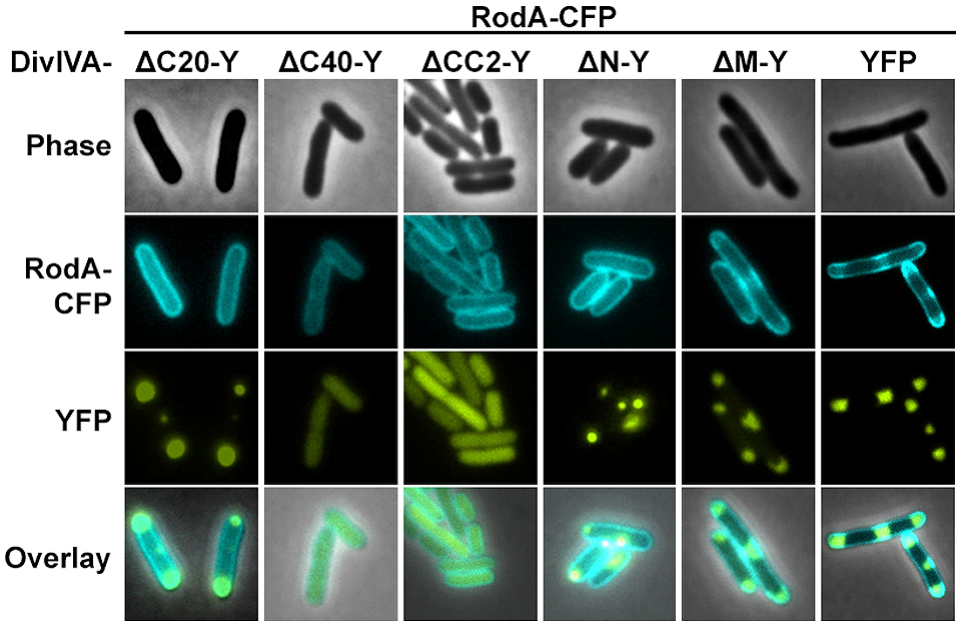
**Co-localization studies of DivIVA-YFP mutants with RodA-CFP from *C. glutamicum*.** DivIVAΔC20-YFP is likely non-functional and thus cannot recruit RodA to the cell poles (first column). DivIVAΔC40-YFP and DivIVACC2-YFP, which appear cytoplasmic, cannot be interpreted in terms of co-localization with RodA (second and third column). DivIVAΔN-YFP does not to recruit RodA to the cell poles (fourth column), unlike DivIVAΔM-YFP (fifth column), and full length DivIVA-YFP (sixth column).

**FIGURE 3:**
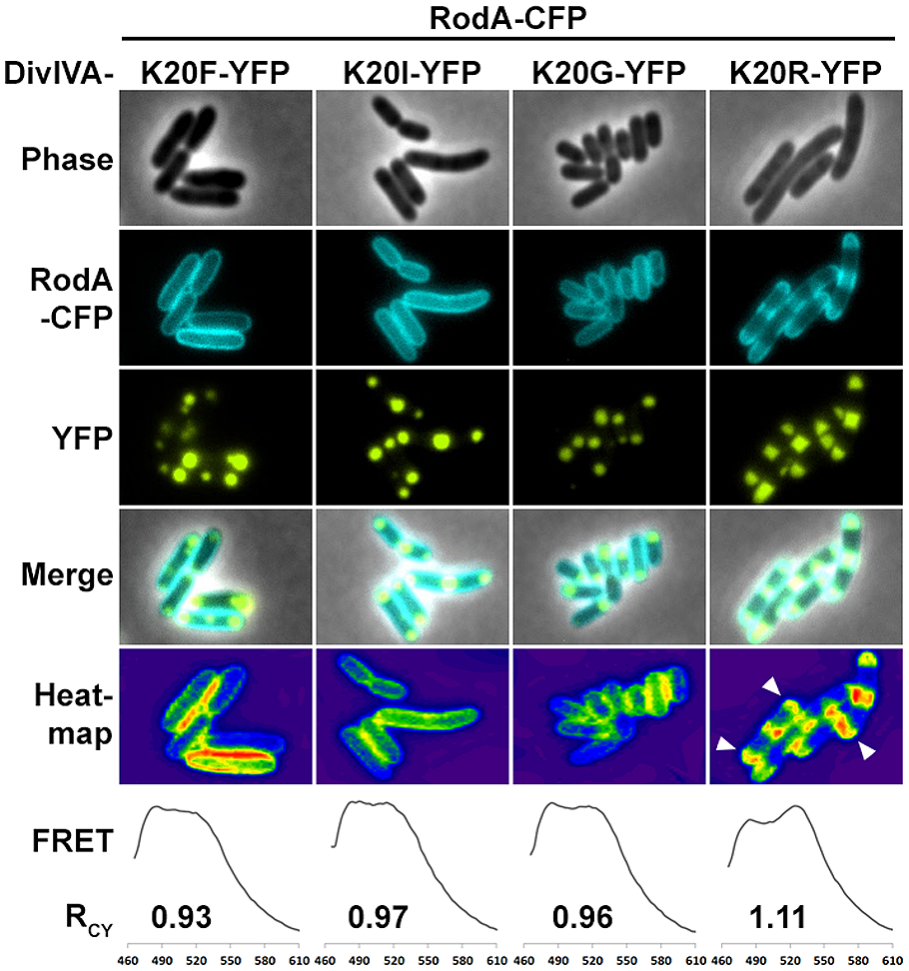
**Fluorescence microscopy of RodA-CFP and DivIVA-YFP mutants K20F-YFP, K20I-YFP, K20G-YFP, and K20R-YFP.** K20F-YFP as well as K20I-YFP and K20G-YFP are not able to recruit RodA to the cells poles. However, the conservative amino acid replacement K20R-YFP allows for DivIVA–RodA interaction. Heat maps emphasize the co-localization (white arrows). R_CY_ values derived from FRET assays describe the ratio of CFP and YFP fluorescence maxima as an indication for FRET efficiency. The R_CY_’s for RodA-CFP with non-interacting DivIVA-YFP mutants are 0.93 (K20F), 0.97 (K20I), and 0.96 (K20G). R_CY_ for interaction between RodA-CFP and DivIVAK20R-YFP is 1.11.

We utilized the established FRET assay to map interaction sites between DivIVA and RodA. Therefore, we co-expressed the DivIVA truncation mutants with full length RodA in *E. coli* and evaluated the interaction microscopically (**Figure 2**). Here we show that DivIVAΔC20 is not able to enrich RodA at the poles. DivIVAΔC40 and CC2 both appear cytoplasmic and consequently do not interact with RodA. Interestingly, DivIVAΔN turned out to be unable to enrich RodA to the cell poles (**Figure 2**, forth column), thereby implicating the involvement of the N-terminal domain for RodA interaction. DivIVAΔM showed the same localization and interaction behavior as full length DivIVA (**Figure 2**, fifth column), thus suggesting that the middle domain is not involved in RodA interaction. We further analyzed the N-terminal domain by mutating the lysine residue at position 20 (K20). When we mutated K20 to a phenylalanine, RodA enrichment was completely abolished (**Figure 3**, left column). The FRET ratio of 0.93 is in the range of approximation without interaction (**Table 3**). Similar, mutations K20I and K20G abolished interaction of DivIVA with RodA (**Figure 3**, middle columns). To address whether the observed loss of interaction depends on the positive charge, we constructed a conservative replacement, K20R. Interestingly, the co-localization of RodA was restored in the K20R mutant, implicating that a positive charge is necessary for interaction with RodA (**Figure 3**, right column, white arrows; R_CY_ value of 1.11). DivIVA variants I18D and I18F did not show any alteration in RodA interaction (Figure S3). Both mutants were able to co-localize RodA-CFP, supporting the notion that not all mutations in that region interfere with RodA interaction.

### RodA’s C-TERMINUS IS INVOLVED IN INTERACTION WITH DivIVA

**FIGURE 4:**
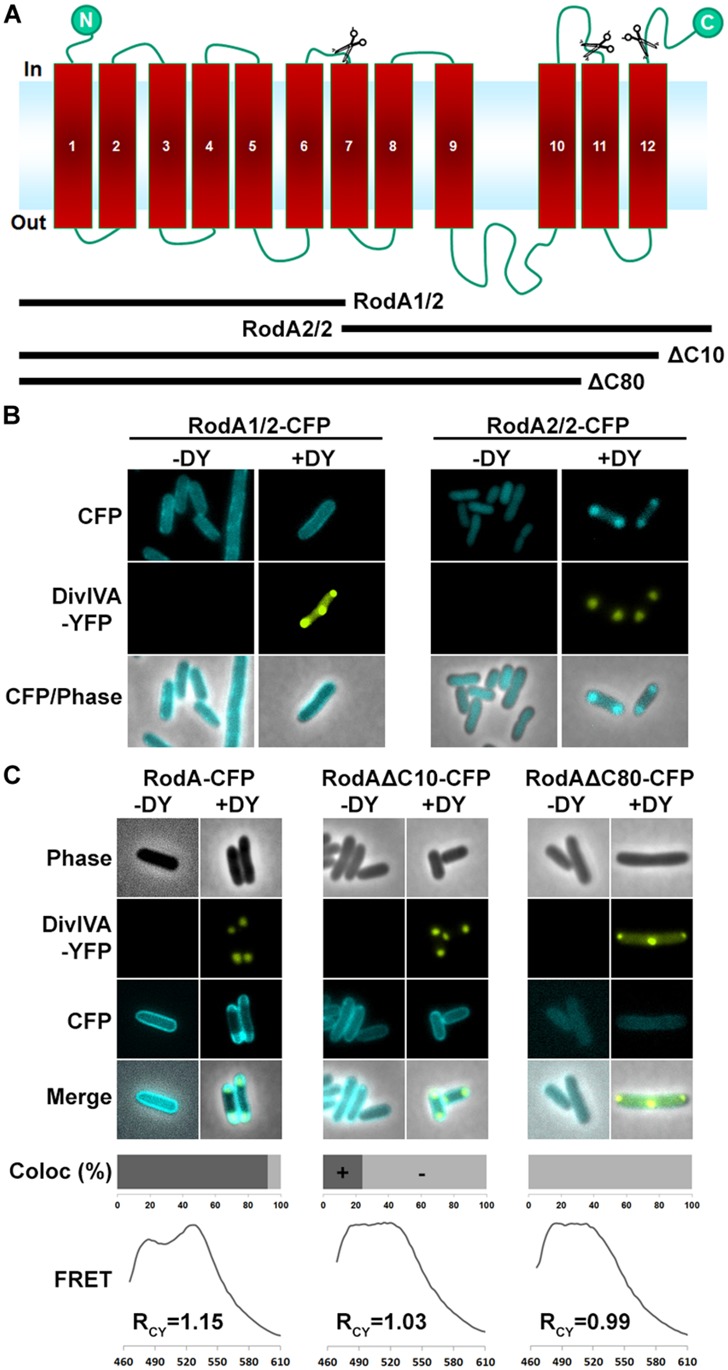
**(A)** Topology model of RodA according to topology prediction (TMHMM). Scissors indicate truncation sites. The protein possesses 12 transmembrane domains and both ends are at the cytoplasmic site. **(B)** Fluorescence microscopy images of full length DivIVA-YFP and the two truncation mutants RodA1/2-CFP and RodA2/2-CFP. While individually expressed RodA1/2-CFP localizes to the membrane (first column), co-localization with DivIVA-YFP seems to be abolished (second column). RodA2/2-CFP lost completely its membrane localization and appears cytoplasmic (third column); however, when co-expressed with DivIVA, it is co-localized to DivIVA foci (forth column). **(C)** Localization of RodA-CFP, RodAΔC10-CFP, and RodAΔC80-CFP (-DY) and co-localization with DivIVA-YFP (+DY). Full length RodA co-localizes to 95% of all DivIVA foci, RodAΔC10-CFP co-localizes to 20%, and RodAΔC80-CFP does not co-localize with DivIVA-YFP. FRET measurements confirm these observations. R_CY_ values are 1.15 for full length RodA, 1.03 for RodAΔC10-CFP, and 0.99 for RodAΔC80-CFP.

**FIGURE 5:**
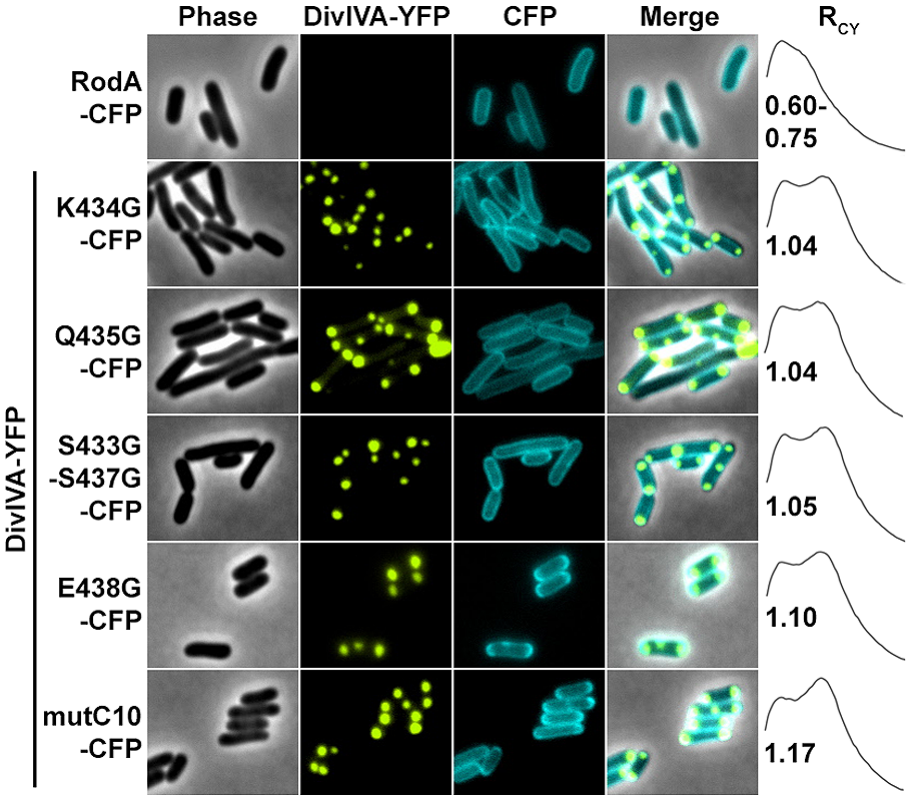
**Fluorescence microscopy images and R_**CY**_ values of RodA-CFP mutants expressed with DivIVA-YFP.** When expressed individually all RodA-CFP point mutants showed membrane localization identical to wild type RodA-CFP, as shown exemplarily in the first row. Co-expression and localization with DivIVA-YFP reveals effects of RodA mutants K434G, Q435G and the double mutant S433G/S437G, implicating that these four AAs are essential for interaction with DivIVA (rows 2–4). Point mutant E438G has no effect on co-localization (fifth row), as well as mutant mutC10 where the last 10 AAs are changed to 10 AAs with similar residues (WT sequence: MSKQASEVAA → AVRNGIADGG). These observations are confirmed by R_CY_ measurements. A positive control with full-length proteins can be found in **Figure 2** and (Sieger et al., 2013).

Next we aimed to identify the RodA interaction site with DivIVA. **Figure 4A** shows a topology model of RodA according to a topology prediction simulation (TMHMM; Arnold et al., 2006). The protein harbors 12 transmembrane domains and both termini are facing the cytoplasm. To identify the interaction site with DivIVA we first divided the protein into two CFP-tagged halves and expressed them individually and together with DivIVA in *E. coli* (**Figure 4B**). It turned out that the N-terminal part (RodA1/2) localized to the membrane, however, it did not co-localize with DivIVA. The C-terminal part (RodA2/2) appeared cytoplasmic, but co-localized completely with DivIVA, implicating that the interaction site must be in the C-terminal half of the protein, although the truncated protein is apparently not inserted correctly into the membrane. We then made CFP-tagged truncations of 10 and 80 AAs from the C-terminus, ensuring cytoplasmic localization of the fluorophore. Whereas >90% of full length RodA-CFP co-localized with DivIVA foci (*R*_CY_ = 1.15), co-localization of RodAΔC10-CFP was reduced to approximately 20% (*R*_CY_ = 1.03) and completely abolished for RodAΔC80-CFP (*R*_CY_ = 0.99; **Figure 4C**). Apparently, the C-terminal 10 AAs contribute to the RodA–DivIVA interaction. We finally tested several point mutations in the C-terminal domain of RodA. We reasoned that maybe a negatively charged residue might interact with K20 that we identified within DivIVA to be responsible for interaction. In spite of this, the variant RodAE438G did not abolish interaction (*R*_CY_ = 1.10). RodAmut10C-CFP, a strain where all C-terminal 10 AAs of RodA were mutated into 10 AAs with similar residues (K → R, Q → N, A → G, *R*_CY_ = 1.17, **Figure 5**) preserved the interaction. However, point mutants K434G, Q435G, and the double mutant S433G–S437G decreased interaction with DivIVA (*R*_CY_ = 1.04, 1.04, and 1.05), implicating an essential role of these four AAs in DivIVA–RodA interaction (**Figure 5**).

**FIGURE 6:**
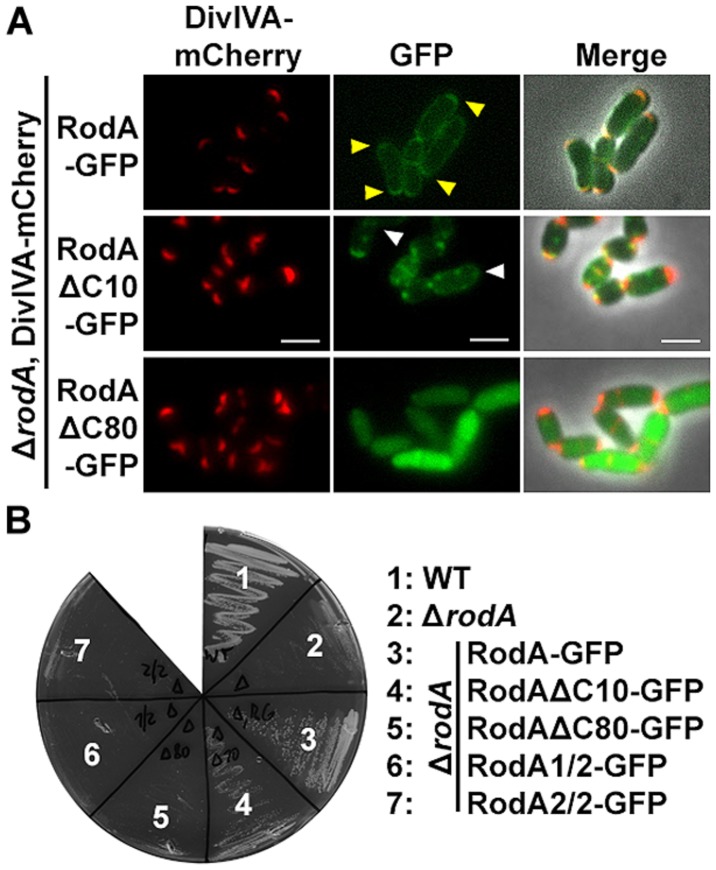
**(A)** Fluorescence microscopy images of *C. glutamicum* DivIVA-mCherry, Δ*rodA* (BSC002), complemented with full length and truncated versions of RodA-GFP. RodA-GFP localizes DivIVA-like to the cell poles (first row, yellow arrows, strain BSC014). RodAΔC10-GFP forms random foci in the cell and several DivIVA foci are free of RodAΔC10-GFP, implicating reduced interaction (second row, white arrows, strain BSC015). RodAΔC80-GFP appears cytoplasmic, polar foci are not observed (third row, strain BSC016). Fluorescence images were taken at equal exposure times (250 ms for mCherry and 500 ms for GFP). Scale bars: 2 μm. **(B)** Growth experiment of WT, Δ*rodA* and complementation strains on Brain Heart Infusion agar. WT and full length complementation showed normal growth after 12 h at 30°C (Sieger et al., 2013). Complementation with RodAΔC10-GFP (strain BSC015) had a slight growth defect, reflecting the observation of reduced DivIVA interaction. Δ*rodA* (BSC001) as well as complementation strains RodAΔC80-GFP (BCS016), RodA1/2-GFP (BSC017), and RodA2/2-GFP (BSC018) could not develop colonies within 12 h.

To support the data obtained with the heterologous expression system, we checked subcellular localization and growth complementation of RodA mutants in *C. glutamicum*. Therefore, we applied fluorescence microscopy after homologous expression of the truncation mutants in Δ*rodA* (**Figure 6A**, strains BSC014-16). In addition we used a DivIVA-mCherry background as topological marker for the cell poles and septa. Whereas full length RodA-GFP localized to the cell poles in a DivIVA-dependent manner (yellow arrows; Sieger et al., 2013), RodAΔC10-GFP localized only to some poles and not always co-localized with DivIVA. Instead, most of the RodAΔC10-GFP formed random foci in the cell that did not co-localize with DivIVA (white arrows). These two observations corroborate the situation in *E. coli*, where only 20% of RodA ΔC10 foci co-localized with DivIVA, implicating loss of tight interaction. RodAΔC80-GFP appeared cytoplasmic and co-localization could not be observed, identical to the situation observed in *E. coli*. These observations were confirmed in a growth experiment on BHI-Agar plates (**Figure 6B**). Wild type and the complementation strain Δ*rodA*/RodA-GFP grew normal, whereas Δ*rodA*/RodAΔC10-GFP showed slight growth defects. All other strains (Δ*rodA*, Δ*rodA*/RodAΔC80-GFP, Δ*rodA*/RodA1/2-GFP, Δ*rodA* RodA2/2-GFP) were not able to support growth within 12 h of incubation at 30°C.

## DISCUSSION

Spatial and temporal organization is a major task in cell cycle regulation of all living species. Topological determinants like DivIVA are involved in spatial regulation of protein machineries such as the Min system in *B. subtilis* and *Listeria monocytogenes* (Bramkamp et al., 2008; Kaval et al., 2014) or the apical growth machinery in *C. glutamicum* (Letek et al., 2008; Sieger et al., 2013). Until now, it was unclear how the apical growth machinery is positioned in actinobacteria. Earlier studies suggested a link between DivIVA and penicillin-binding proteins (Letek et al., 2008), but recently, we could demonstrate the interaction between DivIVA and RodA in *C. glutamicum* (Sieger et al., 2013). However, the protein domains mediating interactions were not mapped. Here, we now identified the N-terminal domain of DivIVA as interaction partner of the C-terminal tail of RodA. We identified a positively charged residue (K20) in DivIVA to play a crucial role in this interaction. Loss of the positive charge at this position abolishes DivIVA–RodA interaction, while the conservative mutation, K20R, restores protein–protein interaction. The N-terminal domain of DivIVA is considered to be important for membrane-binding. The structure of the *B. subtilis* DivIVA reveals that the N-terminus folds into an intertwined-loop which exposes the hydrophobic residue F17. Mutational analysis shows that F17 is essential for membrane binding of the *B. subtilis* DivIVA. Moreover, the binding is backed by positive charged residues (R18; Oliva et al., 2010). Sequence alignments reveal that the N-terminal domain of DivIVA is highly homologous in most Gram positive bacteria. However, residue F17 from *B. subtilis* DivIVA is not conserved in actinobacteria. *Streptomyces*, *Corynebacterium,* and *Mycobacterium* species rather contain a positively charged residue at the corresponding site (**Figure 1A**). The actinobacterial DivIVA homologs contain sequence insertions and the hydrophobic residue mediating membrane association is thus likely I18 in case of *C. glutamicum*. Although mutation of such to an aspartate did not abolish membrane association compared to WT, localization of I18D in the heterologous host is slightly different compared to wild type (**Figure 1C**). Mutation of I18 to a phenylalanine, whose hydrophobic character is even more distinctive, restored polar localization of DivIVA and revealed wild type like membrane association. While membrane binding for the *B. subtilis* DivIVA has been studied in great detail, we know less about membrane binding properties of the actinobacterial proteins. Subcellular localization of *C. glutamicum* DivIVA shows some obvious differences compared to its *B. subtilis* homolog. While the *B. subtilis* DivIVA localizes tightly underneath the polar membrane (Edwards and Errington, 1997), forming a crescent-like structure lining the pole, the *C. glutamicum* protein localizes to the cell poles, but reaches into the cytoplasm (Letek et al., 2009; Donovan et al., 2012), forming a large complex, similar to the PopZ protein found in *C. crescentus* (Laloux and Jacobs-Wagner, 2013). It is therefore likely that membrane binding mechanisms are different in *B. subtilis* and *C. glutamicum* DivIVA proteins. Support to this notion comes from localization studies using truncated DivIVA (DivIVAΔN), lacking the N-terminal domain. Although, DivIVAΔN is significantly more soluble compared to the full length protein, about 64% of the protein is still membrane associated. Despite its role in membrane interaction, the N-terminal domain of DivIVA is essential for interaction with RodA. The positive charge at residue K20 is required for interaction. It is plausible that the exposed, N-terminal loop of DivIVA is required for interaction with a membrane integral protein such as RodA. A central domain of DivIVA has been shown earlier to promote interaction with the origin-binding protein ParB (Donovan et al., 2012), indicating the modular character of DivIVA which encompasses various domains to mediate protein–protein interaction to various partner proteins.

RodA is an integral membrane protein with 12 predicted transmembrane helices. This topology gives rise to several putative binding-sites that could mediate DivIVA binding. A first, rough truncation study where we expressed RodA in two halves indicated that the DivIVA interaction site is likely situated in the C-terminal part of RodA. Further truncation analysis revealed that the last 10 AAs, forming the C-terminal domain facing the cytoplasm are contributing to the RodA–DivIVA interaction. RodAΔC10 has a drastically reduced FRET interaction with DivIVA; however, expression of RodAΔC10 in *C. glutamicum* to some extent complements growth in a Δ*rodA* strain background. Only truncation of the last 80 AAs from the C-terminus completely abolishes localization and interaction with DivIVA. A similar situation has been reported for FtsW, a RodA homolog that is involved in cell division. FtsW interacts via its C-terminal end with FtsZ during cytokinesis of *M. tuberculosis* (Datta et al., 2002). We were able to pinpoint amino acid residues responsible for RodA–DivIVA interaction in RodA (K434G, Q435G, and the double mutant S433G–S437G), suggesting that the RodA C-terminal domain forms an interaction domain which is built by several amino acid residues.

In earlier work we have been showing that ParB interacts with DivIVA in *C. glutamicum*. We have mapped the interaction sites and identified a central region in DivIVA (AAs 144-229) as interaction site with ParB (Donovan et al., 2012). Thus, DivIVA exhibits several exclusive interaction domains allowing DivIVA to act as interaction hub for the connection of apical cell growth and chromosome orientation. The N-terminal region of *B. subtilis* DivIVA interacts with MinJ, a multispan transmembrane protein involved in cytokinesis and division site selection (van Baarle et al., 2013). Thus, DivIVA proteins have evolved to contain interaction motifs for several protein–protein interactions. In actinobacteria DivIVA proteins are essential, likely because they are involved in the spatio-temporal control of two essential cellular processes, cell elongation, and chromosome segregation. Consistent with the fundamental role of DivIVA in these bacteria, actinobacterial DivIVA proteins are larger, containing sequence insertions, when compared to their firmicute counterparts. Future analysis might focus on the regulation of the various protein–protein interactions. DivIVA has been identified as substrate of several protein kinases. Examples of DivIVA phosphorylation have been reported for *M. tuberculosis* (Kang et al., 2005), *Streptomyces coelicolor* (Hempel et al., 2012), and *Streptococcus pneumoniae* (Beilharz et al., 2012). Thereby, phosphorylation of DivIVA has major implications in cell growth or division (Fleurie et al., 2012). It is likely possible that a similar regulatory mechanisms determines chromosome segregation and cell elongation in *C. glutamicum*.

## Conflict of Interest Statement

The authors declare that the research was conducted in the absence of any commercial or financial relationships that could be construed as a potential conflict of interest.
