# Supplementary material for: Interaction sites of DivIVA and RodA from Corynebacterium glutamicum
Source: Front Microbiol. 2015 Jan 7;5:738. doi: 10.3389/fmicb.2014.00738 (PMC4285798; doi:10.3389/fmicb.2014.00738)
Supplement: Supplementary file 1 [file Data_Sheet_1.DOCX]

**Interaction sites of DivIVA and RodA from *Corynebacterium glutamicum***

Boris Sieger and Marc Bramkamp

Ludwig-Maximilians-University Munich, Biocenter, Großhaderner Straße 2-4, 82152 Planegg-Martinsried, Germany

**Supplemental material**

**Table S1: Oligonucleotides**

| **Name** | **Sequence (5’-3’)** | **Restriction site** |
| --- | --- | --- |
| **pETDuet-1** |  |  |
| Div fwd NdeI | CAT CATATG ATGCCGTTGACTCCAGC | NdeI |
| Div Cd20 R | GTA GATATC AG CTGGCCCTCG | EcoRV |
| Div Cd40 R | CAT GATATC TG CGCGATGCGGGTC | EcoRV |
| Div CC1_fwd | GATC CATATG CCTATCGGCAAGCG | NdeI |
| Div CC1 rev +2 | GAT GATATC GG CGCTGCCTGCATG | EcoRV |
| Div CC2 rev +2 | GAT GATATC GG CTCACCAGATGGC | EcoRV |
| Div K20F sense | CTATCGGC **TTC** CGTGGCTACAAC | -- |
| Div K20F antisense | GTTGTAGCCACG **GAA** GCCGATAG | -- |
| Div K20R sense | CTATCGGC **CGT** CGTGGCTACAAC | -- |
| Div K20R antisense | GTTGTAGCCACG **ACG** GCCGATAG | -- |
| RodA 1/2 R | GATA GTCGAC GCCGAGGAAACG | SalI |
| RodA 2/2 F | CGCG GAATTC G ATGGATTTCCCTCG | EcoRI |
| RodA dC80 R | GATA GTCGAC CAGGGTAGCGGTG | SalI |
| RodA dC10 R | GATT GTCGAC TGC GACAGGTCGG | SalI |
| RodA mutC10 Sal R | GATA GTCGAC **ACC TCC ATC TGC GAT ACC ATT TCG TAC TGC** GAC AGG TCG GCG GGC | SalI |
| RodA E438G R | GATA GTCGAC CGC AGC CAC **TCC** CGA TGC TTG CTT G | SalI |
| RodA K434G R | GATA GTCGAC CGC AGC CAC CTC CGA TGC TTG **TCC** GGA CAT GAC AGG | SalI |
| RodA Q435G R | GATA GTCGAC CGC AGC CAC CTC CGA TGC **TCC** CTT GGA CAT GAC AGG | SalI |
| RodA S433G S437G R | GATA GTCGAC CGC AGC CAC CTC **TCC** TGC TTG CTT **TCC** CAT GAC AGG TCG | SalI |
| Div K20G sense | CTATCGGC **GGT** CGTGGCTACAAC | -- |
| Div K20G antisense | GTTGTAGCCACG **ACC** GCCGATAG | -- |
| Div K20I sense | CTATCGGC **ATA** CGTGGCTACAAC | -- |
| Div K20I antisense | GTTGTAGCCACG **TAT** GCCGATAG | -- |
| Div I18F sense | GCCGCCT **TTC** GGCAAGCG | -- |
| Div I18F antisense | CGCTTGCC **GAA** AGGCGGC | -- |
| Div I18D sense | GCCGCCT **GAT** GGCAAGCG | -- |
| Div I18D antisense | CGCTTGCC **ATC** AGGCGGC | -- |
| **pEKEX2** |  |  |
| 12+RodA SalI F | GATC GTCGAC TTGTAGGGAGGTCTC | SalI |
| RodA SacI ns R | AAT GAGCTC CGCAGCCACCTCCGAT | SacI |
| RodA 1/2 R SacI ns | GATA GAGCTC GCCGAGGAAACG | SacI |
| RodA 2/2 F SalI | GATC GTCGAC ATGGATTTCCCTC | SalI |
| RodA dC80 R SacI ns | GATA GAGCTC CAGGGTAGCGGTG | SacI |
| RodA dC10 R SacI ns | GATT GAGCTC TGC GACAGGTCGG | SacI |

**Figure S1**

Visualization and FRET of untagged fluorophores CFP and YFP (top) and RodA-CFP and DivIVA-YFP (bottom) in *E. coli* cells. Under conditions of protein overexpression (1mM IPTG) and a homogeneous protein distribution of CFP and YFP in the cytoplasm, no FRET signal is generated. In comparison, RodA-CFP and DivIVA-YFP generate a FRET signal.

**Figure S2**

Co-localization and FRET measurements of DivIVA and ParB were done according to Donovan et al. (2012) and Sieger et al. (2013) and serve as control experiment for this assay. DivIVA and ParB have been reported to interact during chromosome segregation in *C. glutamicum* and serve as positive control. A mutation of the conserved arginine in position 21 in ParB, resulting in ParBR21A, has been identified to prevent interaction with DivIVA and thus serves as negative control. The FRET values are 0.57 for CFP fluorescence, 1.16 for ParB-DivIVA interaction and 0.95 for ParBR21A-DivIVA approximation. FRET values of the interacting and non-interacting proteins can also be found in our classification of FRET ratios in table 1.

**Figure S3**

Co-localization of DivIVA mutants DivIVA I18D and I18F with RodA-CFP reveal that both DivIVA variants are able to co-localize RodA to the cell poles. I18 has no influence on the co-localization ability of DivIVA. FRET values are 1.14 for I18D and 1.27 for I18F.
