# Supplementary figures and images for: Interaction sites of DivIVA and RodA from Corynebacterium glutamicum
Source: Front Microbiol. 2015 Jan 7;5:738. doi: 10.3389/fmicb.2014.00738 (PMC4285798; doi:10.3389/fmicb.2014.00738)

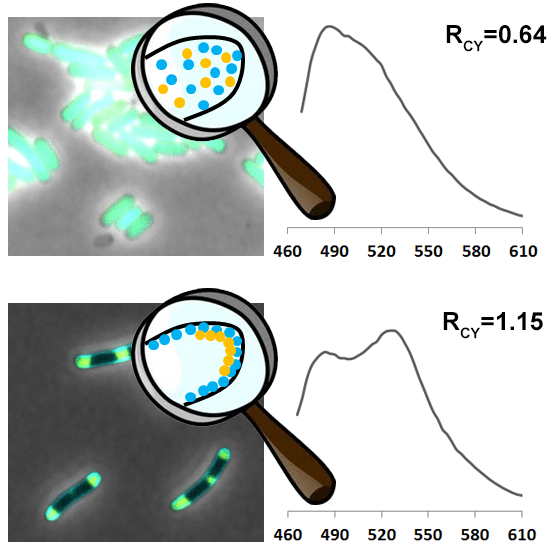

Supplement: Supplementary file 2 [file Image_1.TIF]

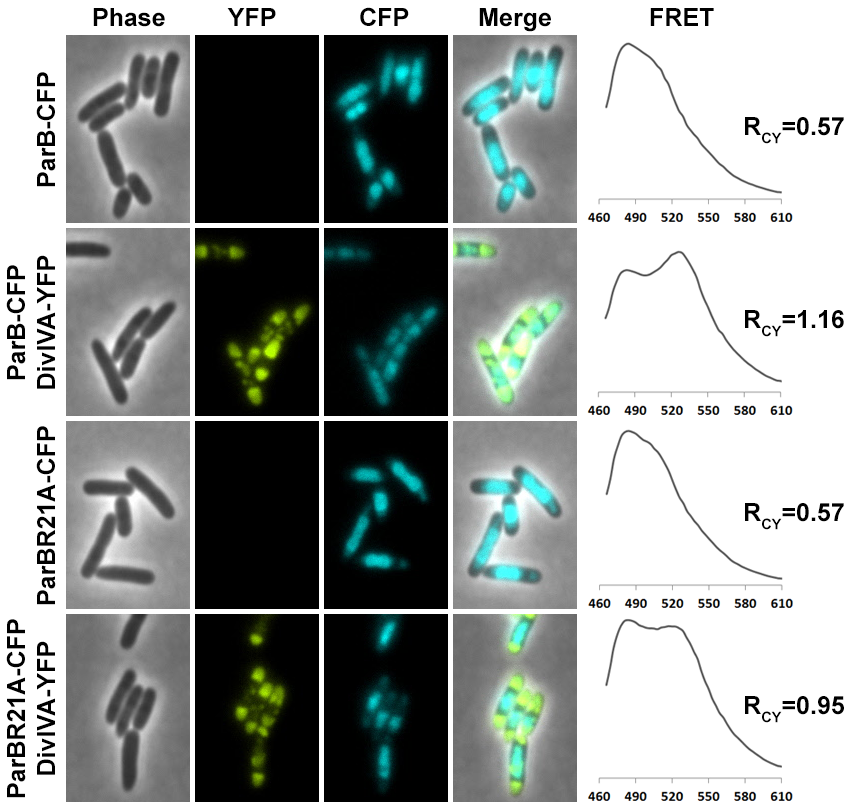

Supplement: Supplementary file 3 [file Image_2.TIF]

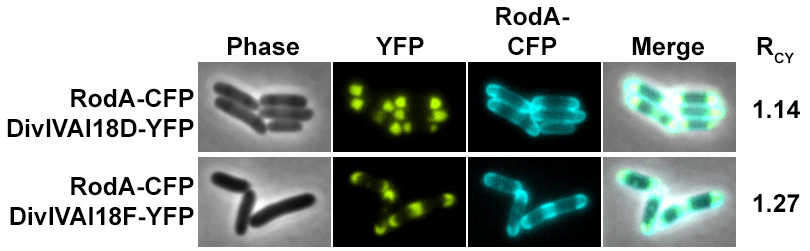

Supplement: Supplementary file 4 [file Image_3.TIF]
